# Treatment of Basal Cell Carcinomas with Pulsed Dye Laser: A Case Series

**DOI:** 10.1155/2012/286480

**Published:** 2012-12-13

**Authors:** Norman Minars, Marianna Blyumin-Karasik

**Affiliations:** ^1^Minars Dermatology Skin and Laser Center, 4060C Sheridan Street, Hollywood, FL 33021, USA; ^2^Department of Dermatology and Cutaneous Surgery, Voluntary Faculty, University of Miami Hospital, 1295 NW, 14th Street, South Building, Suites K-M, Miami, FL 33136, USA

## Abstract

*Background*. Basal cell carcinoma (BCC) is the most prevalent skin cancer. Because of its highly vascular characteristic, it is amendable to treatment with pulse dye laser (PDL). The goal of this study is to determine the safety and efficacy of PDL therapy for mostly facial BCCs. *Materials and Methods*. Sixteen men and thirteen women (29 total) with 39 biopsy-proven BCCs were treated with 1–4 PDL (595 nm) therapies at 2–4-week intervals. The treatment parameters included pulse energy of 15 J/cm 2, pulse length of 3 millisecond, with no dynamic cooling, and 7 mm spot size. The age of the patients was 30–90 years (mean 73 years). Response rates were evaluated by the clinical assessments with mean followup of 11 months. *Results*. Twenty-four patients with thirty-two tumors reached at least three months followup: 24/32 (75%) tumors with complete resolution (mean 3 treatment sessions); 5/32 (16%) tumors recurred; 3/32 (9%) tumors with incomplete responses after four treatments. Minimal side effects and discomfort were experienced by the patients with PDL therapy. *Conclusion*. PDL is a safe, tolerable, and moderately effective method of treating various BCCs. The ideal niche and standardized settings for PDL treatment of BCCs are yet to be determined.

## 1. Introduction

Basal cell carcinoma (BCC) is the most prevalent skin cancer in the world, and its prevalence is ever increasing. Although metastatic rate of BCC is very low, if left untreated, it can cause significant morbidity such as anatomic disfigurement. BCC most commonly affects older light-skinned adults.

Currently, there are numerous options available to treat BCCs including surgical, destructive, laser, X-ray, intralesional chemotherapy, and topical therapies [1, 2]. Each of these treatments has its own risks and benefits as well as varying cure rates. While considering location, size, type, and number of tumors, it is the goal of the dermatologist together with the patient to determine the optimal treatment for the BCC. Although they carry high rates of efficacy, ablative procedures that treat BCCs can be time-consuming, costly, and can lead to infections and scarring [2]. Nonablative topical treatments, such as chemotherapy and immunomodulators, can be cumbersome and unreliable when used by patient at home. These topical treatments are also limited by their primary efficacy in smaller superficial BCCs and higher recurrence rates then surgical therapies [3]. Elderly patients are often diagnosed with multiple BCCs, many of which are in cosmetically important areas, and have other medical comorbidities which may contraindicate surgical treatment modalities (such as blood coagulopathies, slowly healing wounds, and disability that prevents proper wound care). Consequently, there is a need for more efficient, minimally invasive, tolerable, and aesthetically pleasing method to treat BCCs. The use of laser therapeutics to treat BCCs has potential to address this need. Pulsed dye laser therapy is one of the Nonablative treatments of BCCs that we are currently investigating. 

Several reports have described the use of pulsed dye laser (PDL) for the treatment of BCCs [4–10]. Shah et al., Campolmi et al., Tannous et al., Tran et al., and Konnikov et al. have demonstrated safety and efficacy of using PDL to treat BCCs [4–8]. PDL is generally used to treat vascular skin lesions (e.g., port-wine stains, hemangiomas, and telangiectasias). BCCs are highly vascular tumors with clinically apparent telangiectasias. Although exact mechanism of action of using PDL to treat BCCs is unclear, the main hypothesis is antiangiogenic, via specific targeting of the numerous dilated blood vessels found in BCCs, PDL destroys the tumor's bloods supply, leading to eventual demise of the tumor [4, 8]. We believe that PDL therapy of BCCs offers a beneficial therapeutic alternative because it is less invasive and destructive, quick, and simple, especially for patients with disabilities, multiple BCCs, coagulopathic disorders, and concerns about aesthetic results. In this paper we will present the results of our case series treating a range of BCCs with PDL.

## 2. Materials and Methods

Sixteen male and 13 female patients with 39 biopsy-proven BCCs were recruited from our private outpatient population from July 2009 to July 2010. The patients who preferred to avoid surgical intervention of BCC(s) were offered recruitment. The age of the patients was 30–90 years (mean 73 years). All 39 tumors received one to four (1–4) PDL treatments at 2–4-week intervals. At the first visit, the presumed BCC was shave biopsied and pathologically confirmed. Within two weeks of biopsy results, the patients returned to the office and were presented with treatment options. After explaining to the patients the off-label and unconventional use of PDL to treat BCCs, patients gave informed consent to participate in the case series. This study was conducted according to Good Clinical Practice Guidelines and the principles of the Declaration of Helsinki.

On the same visit, after photography, the BCC(s) were treated with a 595 nm pulsed dye laser (PDL) (VBeam, Candela Corporation) using the following parameters: one pass at energy of 15 J/cm^2^, pulse length of 3 ms, no cooling, 7 mm spot size, and 25% overlapping between the pulses with 3–5 mm margins included. The treatment site developed a dusky blue discoloration after each treatment. These parameters were selected based on previously reported similar studies [4, 5]. If the patients complained of significant pain during the first treatment session, local anesthesia with intralesional 2% lidocaine and epinephrine was injected prior to subsequent treatments. Each patient underwent 1–4 treatments (average being three sessions), based on theinvestigator's judgment of clearance. The treatment endpoint (complete clearance) was judged on the absence of clinical evidence of the original BCCs. Patients returned for follow-up visits with clinical assessment and posttreatment photographs, 3–25 months (average being 11 months) after the final treatment. 

## 3. Results

PDL was well tolerated by all of the patients with minimal short-term pain (few minutes) during procedure. Only few patients requested local anesthesia on follow-up treatments (about 1/3 of the patients and this did not correlate with treatment response). Typical side effects after procedure included erythema, mild edema, and dusky purpura, which subsided in one week. Three patients experienced erosions with crusting that resolved without secondary infection or bleeding in two weeks with proper wound care (e.g., topical triple antibiotic or petrolatum ointment twice daily). Three patients developed minor postinflammatory hypopigmentation seen on the 3-month follow-up visit. Scarring was not seen in any of the treated patients. Overall, all of the patients were pleased with the satisfactory cosmetic results. 

**Figure 1:**
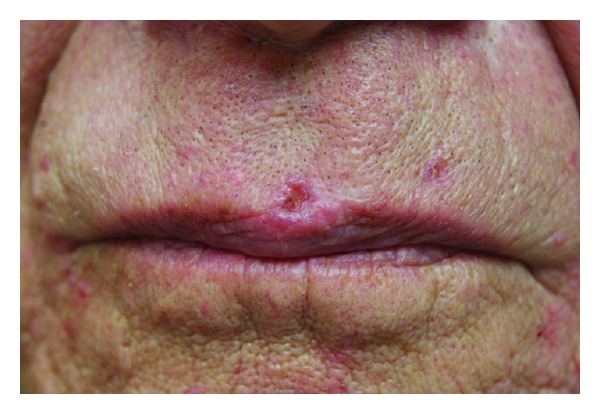
BCCs on the upper mid lip before PDL treatment.

**Figure 2:**
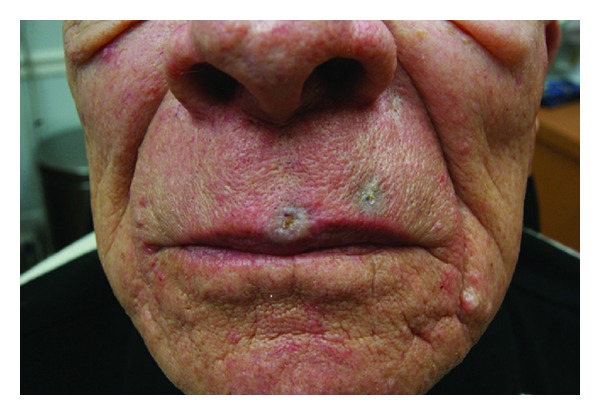
BCCs on the upper mid lip immediately after PDL treatment.

**Figure 3:**
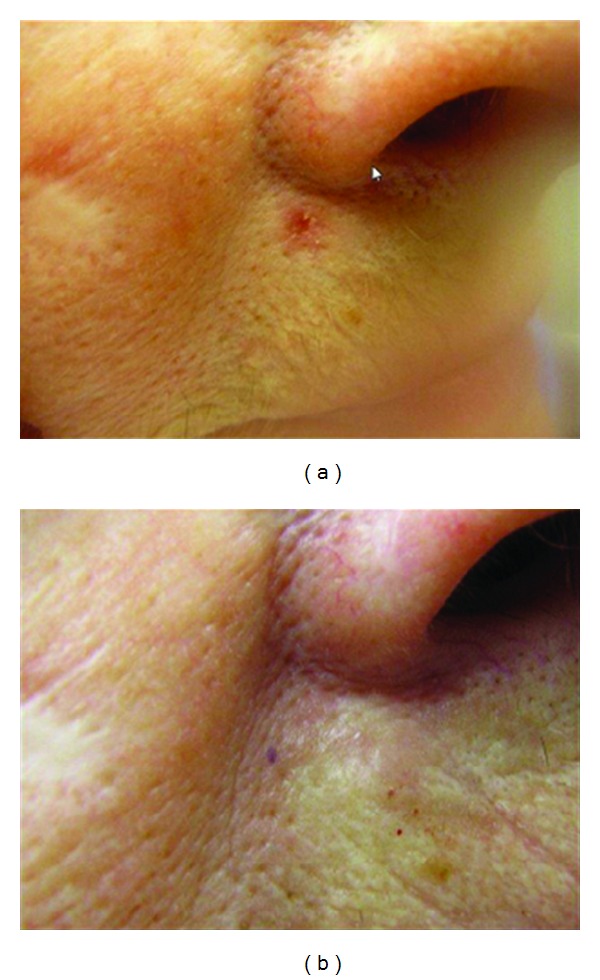
BCC in the right upper lip, before and three months after PDL treatment. Complete resolution of the lesion.

**Figure 4:**
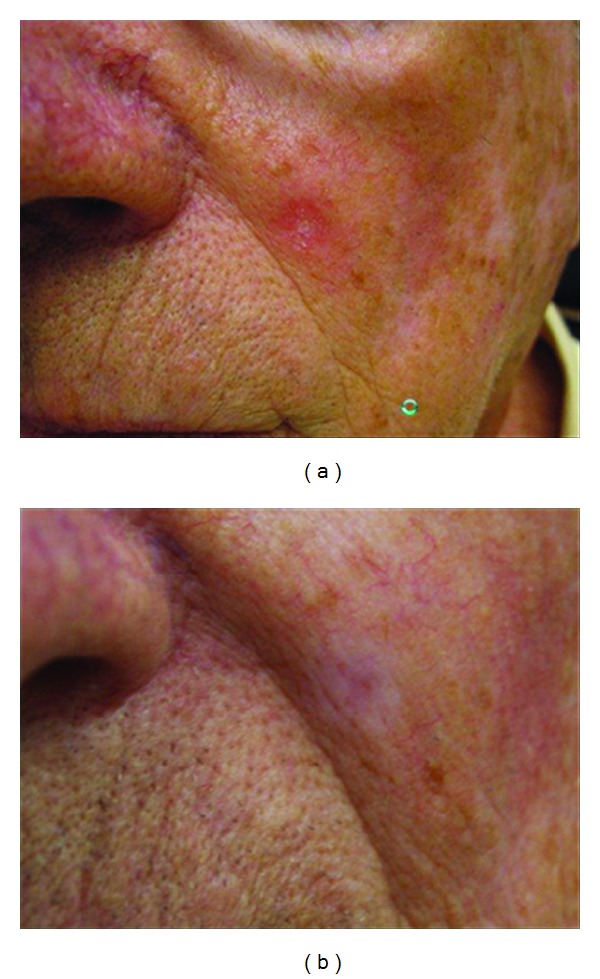
BCC on the left cheek, before and three months after PDL treatment. Complete resolution of the lesion.

The clinical features and response rate of BCCs included in the study are exhibited in Table 1. Although the size of tumors was not recorded, the majorities of tumors were approximately 0.5 cm in diameter and located on the face. The subtypes of BCC were nodular, micronodular, superficial, infiltrative, ulcerative, sclerotic, pigmented, keratinized, and mixed. Most frequent BCC subtypes were nodular and mixed. Treatment response was based on clinical assessment. Most of the patients declined follow-up skin biopsy for cosmetic reasons. Twenty-four patients with thirty-two tumors reached at least three months followup: 24/32 (75%) tumors with complete resolution (mean three treatment sessions); 5/32 (16%) tumors recurred; 3/32 (9%) tumors with incomplete responses after four treatments. Five patients with seven tumors were lost to followup and did not reach three months follow-up period. The tumors that were incomplete or recurred had significant reduction in size based on clinical photographs. All of the tumors that recurred were located on the face. Nodular, infiltrative, or mixed types of tumors were more likely to recur or did not respond to treatment. In general, tumors that underwent three to four therapy sessions had better clearance. Figures 1 and 2 show BCCs before and immediately after the treatment. Lesions achieving complete response are illustrated in Figures 3 and 4.

## 4. Discussion

BCCs are common skin neoplasms that can result in disfigurement and other comorbidities [1]. Even though there are multiple treatments available to treat BCCs, these surgical and nonsurgical therapies have diverse limitations. Ablative therapies Mohs micrographic surgery, and curettage and desiccation are effective with over 90% clearance rates [2]. However, they can be time-consuming, expensive, and fraught with potential side effects [2]. Nonsurgical treatments, chemotherapy, immunomodulator topical therapies, and photodynamic therapy have less risky side effects [3]. However, they likewise can be time-consuming, bothersome to apply, uncomfortable, and deliver lower and unreliable efficacy rates based on application compliance and subtypes of treated BCCs [3]. Therefore, there is currently a search for unconventional, effective, safe, tolerable, and affordable therapy, such as laser therapy. Our case series demonstrates usefulness of PDL therapy for BCCs.

Shah et al. reported a safe and effective use of PDL to treat BCCs [4]. They used settings of one pass, 15 J/cm^2^, 3 ms, no cooling, and 7 mm spot size with 10% overlap; these settings were also used in our study. In this controlled study, twenty BCCs were treated every 2 weeks with PDL for total of four treatments [4]. Shah et al. reported 91.7% of smaller (<1.5 cm) BCCs on trunk and extremities resolved with PDL therapy [4]. Our clinical response rate is lower at 75%; this difference may be due to more varied BCC types, tumor sizes, and facial locations and different number of treatment sessions (1–4) performed in our study. Furthermore, Shah et al. did not have long-term follow-up results which may have altered their clearance rate. Interestingly, Compolmi et al. successfully treated 20 superficial facial and truncal BCCs with PDL with similar treatment parameters (only difference being 1.5 ms pulse duration) and similar clearance rate of 80% [5]. In this study, five treatments were completed within a month and followup was 6–12 months; so, their slightly higher response rate is maybe due to more treatments done for BCCS or lower pulse duration. Another investigative study performed by Tannous et al. with similar treatment settings as ours (only difference being the use of dynamic cooling 30/20) treated 20 superficial and nodular BCCs on trunk and extremities with 92% clearance [6]. Tannous et al. reported an excellent tolerance and cosmetic result after 3–5 treatments at 2–4 week intervals. The difference between their and our clearance rates may be associated with more treatments (mean 4) performed as well as their tumor locations limited to trunk and extremities versus ours on trunk and face. This study was expanded by Konnikov et al. with longer follow-up of one year to demonstrate a 90% clearance rate [8]. It is advantageous to use dynamic cooling during PDL therapy to minimize procedural discomfort and potential surrounding tissue and/or epidermal damage. However, we were hesitant to use dynamic cooling in our study due to concern that it may alter depth of energy delivery and diminish outcomes. However, since Konnikov et al. demonstrated that dynamic cooling does not affect treatment efficacy, it is recommended to use this feature in future PDL therapy of BCCs [8]. 

Allison et al. and Ballard et al. utilized PDL to treat BCCs with poor outcomes [9, 10]. Since both studies used only one treatment, it was recommended to utilize multiple one-pass treatments to achieve better outcome as seen in previously discussed studies [4–6, 8]. Diversely, a recent study by Tran et al. used pulse stacking, one-treatment technique with PDL to treat BCCs and had better outcomes [7]. Tran et al. used similar laser settings as in our study (except using 7.5 J/cm^2^ energy and 10-mm spot size and double-stacking pulses). They treated different subtypes of BCCs and squamous cell carcinomas on the trunk and extremities with 71% clearance rate [7]. The study was controlled, and efficacy was confirmed histopathologically without long-term follow-up assessment [7]. This clearance rate is similar to our study, and the adverse effects of few cases of hypopigmentation and mild scarring were similar to our study and other studies conducting multiple treatments of BCCs [4–6, 8]. One-time treatment would be more beneficial since it is more convenient and less costly to the patient. However, since other studies did have higher clearance rate, it is difficult to say whether one-time stacking treatment with PDL of BCCs is preferred over multiple treatments approach [4–6, 8]. Further comparison studies are required to elucidate this issue.

In our study, we treated varied (e.g., nodular and mixed) BCC subtypes and also included treatment of facial tumors. Based on our observations, histology types of the tumors are likely to affect the outcome, where nodular, infiltrative, and mixed tumor types were more likely to recur after PDL therapy. Also notable is the rapid period for recurrence in four of our cases, which was about 3 months. These cases may represent incomplete clearance versus recurrence, something that could be determined with followup histology, which was not done in this study. These findings will need to be evaluated and confirmed in future studies. 

PDL offers several benefits over conventional ablative therapies, such as surgery and curettage and desiccation. PDL is more time-efficient, simple to perform, has short recovery time, as well as carries less risk of side effects commonly associated with surgical or ablative therapies (e.g., bleeding, scarring, poor wound healing, and secondary infections). Although no scoring system was used to assess these parameters, based on our impression, majority of our patients were pleased with safety, tolerability, and efficacy of the laser treatment. Based on the findings in our study and previous studies using PDL therapy for BCCs, it is a satisfactory cosmetic treatment option. Although the response rate of BCCs to PDL therapy may be variable, it is still important to note that the treatment does reduce the size of the tumors which is helpful for patients that are interested in pursuing further alternative treatment options (e.g., Mohs surgery), especially in cosmetically pertinent areas [4, 5, 8]. On the other hand, PDL treatment of BCCs also has its drawbacks, such as lack of universal access to laser therapy, requirement of several in-office treatments, and lower response rate compared to more traditional therapies (e.g., surgical and destructive methods), as seen in our study.

This study is a case series and, consequently, has some limitations. It was conducted with a small number of patients and with a followup of 3–25 months. Our response rate is also limited by the clinical assessment and absence of control population. Due to the fact that the majority of the study patients declined to have a follow-up biopsy, it was not possible to use standardized pathological confirmation of BCC clearance after PDL therapy. Since shave biopsies were performed for diagnoses of BCCs, these may have influenced clearance results due to potential tumor debulking and inflammatory regression [8]. The treatment parameters for PDL are also not currently standardized, and different pulse durations (0.5–3 ms), fluences (6.5−15 J/cm^2^), and spot sizes (7–10 mm) have been used in past studies [4–10]. Cooling parameters (including no cooling at all)may also affect tolerance and efficacy [4–10]. The ideal setting for PDL treatment of BCC is yet to be determined in future studies. 

Ibrahimi et al. conducted a case study on a patient with basal cell nevus syndrome and treated multiple superficial and nodular BCCs with long-pulsed Alexandrite laser that also has vascular selectivity and deeper tissue penetration [11]. The clearance rate of this study was 83% with seven months followup [11]. Moskalik et al. performed treatment of facial 2,742 BCCs using pulsed Neodymium laser and Nd : Yag laser with 2.2% and 3.1% recurrence rates, respectively [12], with mean followup of 13 months. The efficacy of Nd : Yag laser was attributed to its deep photodestractive mechanism of action that leads to thermal destruction and coagulation of the tissue. The treatment was performed under local anesthesia to minimize discomfort. The majority of the patients had good cosmetic results with minimal scarring. Therefore, Nd : Yag laser maybe another laser therapeutic option for treatment of BCCs.

Our study demonstrates that PDL is a safe and moderately effective alternative treatment for various types of BCCs. Whether PDL can be considered the optimal laser or the optimal Nonablative option for treating BCCs needs to be answered in future well-designed studies. Other parameters, such as cost effectiveness or outcome enhancement with the use of combined therapeutic protocols, need also further evaluation.

## Figures and Tables

**Table 1:** Basal cell carcinoma and response to pulsed dye laser.

Patient	Age	Gender	Lesion	Tumor subtype	Location	Number of treatments	Response	Followup (months)
1	83	M	1	S/N	L cheek	3	C	25
2	73	M	2	N	R cheek	4	C	19
			3	N	L face	4	C	19
			4	N	R jawline	4	C	19
			5	N	Chin	4	C	19
3	53	F	6	N	R forehead	4	C	11
4	60	F	7	N	R shoulder	3	C	21
5	83	F	8	MN	R upper lip	4	C	25
			9	N	L chin	4	C	25
			10	NP	R chin	4	C	25
6	80	M	11	N	L temple	3	L	L
7	85	M	12	K	L forehead	3	L	L
8	85	M	13	N	Mid upper lip	3	C	8
			14	NU	L upper lip	3	C	8
9	82	F	15	NI	R nose tip	4	R	15
10	85	M	16	NI	R nose ala	4	C	5
11	65	M	17	N	Nose tip	3	C	12
12	80	F	18	N	L nose ala	4	C	9
13	63	M	19	NS	L upper lip	4	C	3
14	80	F	20	N	Nose tip	4	Inc	3
15	70	F	21	S/N	Lower lip	3	C	6
16	90	F	22	NU	Nose tip	2	C	25
17	79	M	23	N	Forehead	4	C	3
			24	N	L temple	4	C	3
18	86	M	25	NI	Mid forehead	4	R	3
			26	I	L forehead	4	R	3
19	54	F	27	NI	Nose tip	4	R	3
20	30	F	28	N	L cheek	3	C	6
21	49	F	29	K	L leg	2	L	L
22	79	F	30	S/N	L chin	3	C	5
23	83	M	31	NS	Scalp	4	Inc	3
24	81	M	32	NI	Mid upper lip	4	R	3
25	48	M	33	N	R shoulder	4	Inc	3
26	79	M	34	N	R chin	3	L	L
			35	N	L nose	3	L	L
			36	N	L jawline	3	L	L
27	90	F	37	S	L nose ala	3	L	L
28	72	M	38	U	L helix ear	1	C	4
29	81	M	39	N	Nose tip	1	C	18

Pathologic subtypes. S: superficial; S/N: superficial-nodular; N: nodular; MN: micronodular; K: keratinized; NP: nodular-pigmented; NU: nodular-ulcerative; NI: nodular-infiltrative; NS: nodular-sclerotic; I: infiltrative; U: ulcerative.

Response. C: complete with at least 3 months followup evaluation; Inc: incomplete response; L: lost to followup; R: recurrence.
